# Optimal Regimens and Clinical Breakpoint of Avilamycin Against *Clostridium perfringens* in Swine Based on PK-PD Study

**DOI:** 10.3389/fphar.2022.769539

**Published:** 2022-02-24

**Authors:** Anxiong Huang, Xun Luo, Zihui Xu, Lingli Huang, Xu Wang, Shuyu Xie, Yuanhu Pan, Shiwei Fang, Zhenli Liu, Zonghui Yuan, Haihong Hao

**Affiliations:** ^1^ National Reference Laboratory of Veterinary Drug Residues (HZAU) and MOA (Ministry of Agriculture) Key Laboratory for Detection of Veterinary Drug Residues, Wuhan, China; ^2^ MOA Laboratory for Risk Assessment of Quality and Safety of Livestock and Poultry Products, Wuhan, China

**Keywords:** *Clostridium perfringens*, avilamycin, optimal regimens, clinical breakpoint, PK-PD

## Abstract

*Clostridium perfringens* causes significant morbidity and mortality in swine worldwide. Avilamycin showed no cross resistance and good activity for treatment of *C. perfringens*. The aim of this study was to formulate optimal regimens of avilamycin treatment for *C. perfringens* infection based on the clinical breakpoint (CBP). The wild-type cutoff value (CO_WT_) was defined as 0.25 μg/ml, which was developed based on the minimum inhibitory concentration (MIC) distributions of 120 *C. perfringens* isolates and calculated using ECOFFinder. Pharmacokinetics–pharmacodynamics (PK-PD) of avilamycin in ileal content were analyzed based on the high-performance liquid chromatography method and WinNonlin software to set up the target of PK/PD index (AUC_0–24h_/MIC)_ex_ based on sigmoid E_max_ modeling. The PK parameters of AUC_0–24h_, C_max_, and T_max_ in the intestinal tract were 428.62 ± 14.23 h μg/mL, 146.30 ± 13.41 μg/ml,, and 4 h, respectively. The target of (AUC_0–24h_/MIC)_ex_ for bactericidal activity in intestinal content was 36.15 h. The PK-PD cutoff value (CO_PD_) was defined as 8 μg/ml and calculated by Monte Carlo simulation. The dose regimen designed from the PK-PD study was 5.2 mg/kg mixed feeding and administrated for the treatment of *C. perfringens* infection. Five respective strains with different MICs were selected as the infection pathogens, and the clinical cutoff value was defined as 0.125 μg/ml based on the relationship between MIC and the possibility of cure (POC) following nonlinear regression analysis, CART, and “Window” approach. The CBP was set to be 0.25 μg/ml and selected by the integrated decision tree recommended by the Clinical Laboratory of Standard Institute. The formulation of the optimal regimens and CBP is good for clinical treatment and to control drug resistance.

## 1 Introduction

*Clostridium perfringens* is a common cause of intestinal diseases in humans, animals, fish, and their environment. It has caused serious damage to the global economy in the last few years (Allaart et al., 2014; Nan et al., 2017; Yadav et al., 2017; Yan et al., 2017). *C. perfringens* can be divided into five types, including A, B, C, D, and E types, based on the differences between its toxigenic genes and pathogenicity (Hatheway 1990). *C. perfringens* diseases in pigs are generally caused by type A and type C (Lee et al., 2014). Type A is often linked to diarrhea in suckling piglets with mild necrotizing enterocolitis (Springer et al., 2012); hemorrhagic necrotic enteritis is induced by type C in piglets aged 0–2 weeks (Waters et al., 2003; Nan, Hao et al., 2017).

Avilamycin is an orthosomycin family antibiotic derived from the fermentation products of *Streptomyces viridochromogenes* (Mertz et al., 1986; Saito-Shida et al., 2018), including the major active factor avilamycin A and 15 other small factors (JECFA, 2004). Avilamycin is one of the EU-approved antimicrobial agents in the feed industry and is currently only used in animals to control bacterial enteric infections and multidrug-resistant gram-positive bacteria (Ho et al., 2013; Lv et al., 2013; OIE, 2015). Avilamycin has a strong treatment efficacy against necrotic enteritis (Vissiennon et al., 2000; Paradis et al., 2016). It does not display cross-resistance with any other antimicrobial agents, suggesting that this type of antimicrobial agents may represent an avenue for the for development of new antimicrobial agents (Aarestrup et al., 2000; Weitnauer et al., 2001; Arenz et al., 2016; Krupkin et al., 2016). It can be placed at the frontline of drugs due to its low environmental toxicity, extensive metabolism *in vivo*, and reduced ecological hazards (Arenz, Juette et al., 2016; Krupkin, Wekselman et al., 2016).

In order to reduce the occurrence of drug resistance, a reasonable dosing regimen is necessary. Pharmacokinetics—pharmacodynamics (PK-PD) study are very important for developing a reasonable dosing regimen (Yoshii et al., 2016). Many PK-PD studies mainly focus on the analysis of PK data in the plasma, because the data in the plasma is relatively stable and easy to obtain, but the Clinical Laboratory of Standard Institute (CLSI) also mentions that when conditions permit, the PK of target tissues can also be used (CLSI 2007a). For infections of *C. perfringens*, the drug concentration in the ileal contents (an unformed stool sample taken from the ileum) should be used. Compared with disease animal models, healthy animal models have better stability and reproducibility, which is even more important (CLSI 2007a).

The clinical breakpoint (CBP) is of great significance to antimicrobial susceptibility testing and monitoring of resistance (OIE 2016; Toutain et al., 2017; Lei et al., 2018). The determination of interpretive CBPs needs a comprehensive analysis of relevant information, including wild-type cutoff value (CO_WT_)/epidemiological cutoff value, PK-PD cutoff value (CO_PD_), and clinical cutoff value (CO_CL_). The CO_WT_ needs large numbers of *in vitro* minimum inhibitory concentrations (MICs) or zone diameter tests (Turnidge et al., 2007), and it allows the detection of resistance as a biological phenomenon (Toutain, Bousquet-Mélou et al., 2017). The CO_PD_ is the relationship between drug concentrations and microbial PK parameters *ex vivo* (Kahlmeter et al., 2003; CLSI 2007a). The CO_CL_ is the clinical outcomes with MIC from prospective clinical studies (Ambler et al., 2008). However, the clinical data is not easy to achieve from current studies; the CO_WT_ could be used to separate susceptible isolates from isolates with resistance when there is a lack of clinical data (Espinel-Ingroff et al., 2011; Peng et al., 2016). But the CO_WT_ cannot represent the CO_PD_ and clinical efficacy (Barbour et al., 2010). When the three cutoff values are established, the decision tree from CLSI is applied for establishing the CBPs (CLSI, 2007a).

The purpose of the current study is to formulate the optimal regimens of avilamycin treatment for *C. perfringens* infection based on the CBP, and the CBP has been based on the CO_WT_, CO_PD_, and CO_CL_ of avilamycin against *C. perfringens*.

## 2 Materials and Methods

### 2.1 Chemicals and Reagents

Avilamycin premix (10%) was obtained from Eli Lilly, United States. Avilamycin (90%) was separated at the Institute of Veterinary Pharmaceuticals (Wuhan, China) and was confirmed by high-performance liquid chromatography (HPLC) and liquid chromatograph-mass spectrometer/Ion trap/Time of flight. All chemicals used in this experiment were of analytical or higher grade.

### 2.2 Bacterial Isolates

The clinical *C. perfringens* isolates were isolated from anal swab samples from piglets with or without diarrhea from large swine farms in the Henan, Hubei, Jiangxi, Liaoning, and Hunan Provinces of China. 120 positive strains were identified based on multiplex polymerase chain reaction. All bacteria were anaerobic cultured on tryptose sulfite cycloserine agar bases containing 5% d-cycloserine. The control strain was *Bacteroides fragilis* ATCC 25285.

### 2.3 Animals

About 74 weaned, castrated, crossbred (Duroc × Large White × Landrace) pigs (15 ± 2 kg) were purchased from the Huazhong Agricultural University's pig farm (Wuhan, China), details of which are as shown in Table 1. Two pigs were used to establish the HPLC method for the detection of avilamycin. Six pigs were used to conduct PK-PD experiments. The other pigs were used for the clinical trials. The pigs were acclimatized for 7 days before the experiment. All the animal experiments were approved by the Animal Ethics Committee of Huazhong Agricultural University (HZAUSW-2015-012) and the Animal Care Center, Hubei Science and Technology Agency, in China (SYXK 2013-0044). All efforts were taken to reduce the pain and adverse effects on the animals.

**TABLE 1:** Experimental grouping of pigs used in the study.

Experiment	Quantity	Use	Strains	MIC (μg/ml)
PK-PD study	2	HPLC	—	—
	6	PK study	—	—
Clinical treatment	6	Blank	—	—
	6	Treatment after infection	HG4	0.015
	6		HN10	0.06
	6		HS42	0.25
	6		HS72	2
	6		HS101	8
	6	No treatment after infection	HG4	0.015
	6		HN10	0.06
	6		HS42	0.25
	6		HS72	2
	6		HS101	8

MIC, minimum inhibitory concentration; PK, pharmacokinetics; PD, pharmacodynamics; HPLC, high-performance liquid chromatography.

### 2.4 Determination of Wild-Type Cutoff Value for Avilamycin Against *C. perfringens*

We used the agar dilution method proposed by CLSI M11-A7, and the interpretation of the results was based on the graphical demonstrations in the CLSI documents (CLSI 2007b).

The CO_WT_ was calculated following the method described by Turnidge et al. (2010), defined as the highest MIC for the wild-type comprising at least 95% of each MIC distribution, according to the CLSI guidelines (Pfaller et al., 2010; Pfaller et al., 2011). Ismail et al. (2018) developed the ECOFFinder program based on this principle, which brought the MIC distributions into the software, automatically fitted, and obtained CO_WT_.

### 2.5 Determination of Pharmacokinetics–Pharmacodynamics Cutoff Value for Avilamycin Against *C. perfringens*

#### 2.5.1 *In Vitro* Pharmacodynamics of Avilamycin Against *C. perfringens* HS42

The strain HS42 near the CO_WT_ using a strong pathogenic test, by mouse virulence test, was chosen to determine the PK-PD study. The MIC of *C. perfringens* HS42 in the fluid thioglycollate medium (FT) and ileal contents was determined according to the broth dilution method recommended by CLSI M11-A7. By detecting the colony-forming unit (CFU) at different time points (0, 1, 2, 4, 6, 8, 12, and 24 h) under different MIC concentrations, we drew the *in vitro* time-kill curve.

#### 2.5.2 High-Performance Liquid Chromatography to Determine Avilamycin Concentration

An Aglient SB-Aq, 250 × 4.6 mm (i.d.), 5-μm column was used for HPLC, which was performed with a 290 nm detection wavelength at 30°C. The mobile phase consisted of 10 nmol/L ammonium acetate (phase A) and acetonitrile (phase B). The plasma was extracted with acetonitrile twice. The ileal content was extracted with acetone twice, and 10% NaCl solution was added with methylene chloride to the extraction. The solution was dried at 50°C under nitrogen. After drying, chloroform was added to dissolve the residue. The silica gel column (Waters, USA) was activated with methanol and chloroform. The dissolving solution was passed through the column to clean, the mixture of chloroform: acetone (4:1) was used to wash, and eluted with a mixture of chloroform: acetone (3:7). The mixture was vortexed and analyzed by liquid chromatography (Sunderland et al., 2004).

The avilamycin standard solution has good linearity in the range of 0.1–20 μg/ml (*R* ^2^ = 0.9997). In the plasma, the detection limit of determination (LOD) was 0.05 μg/ml and the limit of quantitation (LOQ) was 0.1 μg/ml. In the ileal content, the detection LOD was 0.08 μg/ml and the LOQ was 0.1 μg/ml.

#### 2.5.3 Sampling Procedures

A T-shape ileal cannula was installed in the ileum to get the ileal content. Pigs were allowed to recover fully in separate metabolism crates for about 2 weeks and were provided supplemental heat using infrared heating lamps. Before the experiment, food was withheld from the pigs for 36 h and water for 12 h. All the pigs were given 4 mg/kg b.w. of avilamycin by oral administration. At different time points (0, 1, 2, 3, 4, 5, 6, 8, 10, 12, 24, 36, and 48 h), the plasma was collected from the anterior cava, and the ileal content was collected into tubes from the T-shape ileal cannula at the same time. The samples were divided into two aliquots on ice and stored at −20°C for subsequent PK-PD studies.

#### 2.5.4 *Ex Vivo* Pharmacodynamics of Avilamycin Against *C. perfringens* HS42

The *ex vivo* time-kill curves were determined using the ileal content samples obtained from the pigs at different time points after oral administration of 4 mg/kg b.w. avilamycin.

#### 2.5.5 Pharmacokinetics–Pharmacodynamics Integration Analysis

The concentration–time data for avilamycin were analyzed for individual pigs by non-compartmental analysis (WinNonlin; Pharsight Corporation, Mountain View, CA, United States) using the statistical moment approach. The inhibitory sigmoid E_max_ model was used to determine the PD target under different efficiencies [E = 0, −3, and −4 (bacteriostasis, bactericidal, and eradication, respectively)]. The model equation is as follows:

$$E=E_{0}-\frac{PD_{\max}\cdot C^{N}}{C^{N}+EC_{50}^{N}}$$

where E is the summary of PD endpoint and E_0_ is the effect representing the value of the PD endpoint without drug treatment (i.e., the value of the summary endpoint when the PK-PD index is 0). EC_50_ represents (AUC_0–24h_/MIC)_ex_ which produces 50% of the maximum antibacterial effect (PD_max_), C represents the (AUC_0–24h_/MIC)_ex_ ratio, N represents the Hill coefficient, and *PD*_max_ is the maximum effect (in relation to E0) indicated by the plateau where increased exposures result in no further kill.

#### 2.5.6 Monte Carlo Simulation and Pharmacokinetics–Pharmacodynamics Cutoff Value Analysis

Crystal Ball v7.2.2 was used to perform Monte Carlo simulation. The distribution of PK parameter AUC_0–24h_ was assumed to be log-normal. A total of 10,000 subjects were simulated (Andes et al., 2004). The bactericidal effect was selected as a PD target to calculate the probability of target attainment (PTA). The CO_PD_ was defined as the MIC at which the PTA was ≥90%.

### 2.6 Doses Estimation

Assuming PK linearity, the predicted daily doses were calculated by the dose equation (Toutain et al., 2002) as follows:

$$Dose=\frac{{\left(AUC_{0-24h}/MIC\right)}_{ex}\times MIC}{fu}\times CL/F$$

CL/F represents the clearance scaled by bioavailability in ileal content, (AUC_0–24h_/MIC)_ex_ is the targeted endpoint for optimal efficacy in 24 h, MIC is the target pathogen, and fu is the proportion of free drugs in ileal content calculated by equilibrium dialysis.

The balance dialysis method was used to measure the binding rate of avilamycin to the contents of the porcine ileum, and the binding rate was converted into the free drug ratio. The dialysis bag containing blank ileal contents was suspended in dialysate with different drug concentrations and placed at 4°C for dialysis until equilibrium was reached. After the end of dialysis, the samples were taken from inside and outside of the dialysis bag and the drug concentration was determined. The binding rate was calculated based on the concentration. The binding rate = (Dt − Df)/Dt × 100%, where Dt is the drug concentration in the dialysis bag and Df is the liquid drug concentration outside the dialysis bag.

According to the formula of mixed feeding: Dose=d×t/W to decide the group dosage. Where d is the oral dose (mg/kg b.w.), W is the feed intake per 1 kg body weight of pigs for per 24 h, t is the number of oral administration of drugs within 24 h. W was not the same considering the difference in individual animals. For the piglets, the daily intake (24 h) accounts for 4–6% of their body weight (an average of 5%), i.e., 50 g per day per 1 kg of body weight.

The calculated dose, PK parameters, and PD parameters were brought into the Mlxplore software to simulate and predict the growth of bacteria under three doses (preventive dose, therapeutic dose, and eradication dose) and different administration intervals to obtain the best dosing schedule and dosing interval.

### 2.7 Determination of Clinical Cutoff Value for Avilamycin Against *C. perfringens*

#### 2.7.1 Clinical Efficacy by Different Minimum Inhibitory Concentrations

About 66 experimental piglets (15 ± 2 kg) were divided into 11 groups. Each group had six pigs. The type A *C. perfringens*, HG4 (0.015 μg/ml), HN10 (0.06 μg/ml), HS42 (0.25 μg/ml), HS72 (2 μg/ml), and HH101 (8 μg/ml) strains, which have β2 toxin and were chosen by the mouse virulence test, were used to conduct the clinical infected experiment. One group was the blank control group. Five groups were infected with five strains as the negative control groups. Five groups challenged with the five strains were the experimental groups. The negative control groups were infected without administration, the experimental groups were infected by administration, and the administration dose and interval were performed in accordance with the therapeutic administration dose established by the PK-PD study.

#### 2.7.2 Statistical Analysis

Three analytical methods were used, including “Window” approach, CART, and nonlinear regression analysis, to define the relationship between MIC and possibility of cure (POC) and determine the CO_CL_.

The “Window” approach was calculated by CAR and MaxDiff, the cutoff value was between the CAR and MaxDiff when using the “Window” methods (Turnidge et al., 2017). CART was directly simulated by the Salford Predictive Modeler software; the MICs were used as a predictor and the clinical treatment outcomes were the target variable. The NRA was simulated by SPSS software; Log_2_MICs were the independent variable and the POCs were the dependent variable.

### 2.8 Established Clinical Breakpoint

Three cutoff values were analyzed comprehensively using the decision tree proposed by CLSI document M37-A3 to decide the CBP (CLSI 2007a). When CO_WT_ = CO_CL_, CBP is CO_WT_ and CO_CL_; when CO_WT_ > CO_CL_, if CO_PD_ is the smallest, CBP is CO_CL_, otherwise CBP is CO_WT_; when CO_WT_ < CO_CL_, if CO_PD_ is the smallest, CBP is CO_WT_, otherwise CBP is CO_CL_.

## 3 Results

### 3.1 Wild-Type Cutoff Value for Avilamycin Against *C. perfringens*

**FIGURE 1:**
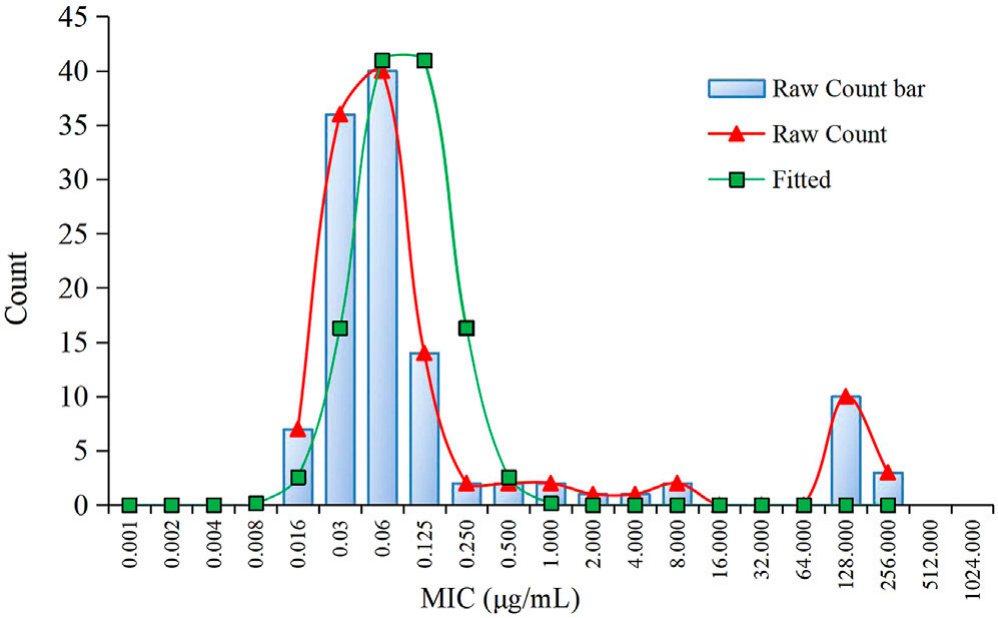
The minimum inhibitory concentration (MIC) distribution of avilamycin against 120 strains of *Clostridium perfringens* and nonlinear regression.

The MIC distributions of avilamycin against the 120 clinical strains with diarrhea of *C. perfringens* type A is shown in Figure 1. The MIC values ranged from 0.015 to 256 μg/ml. The corresponding MIC_50_ and MIC_90_ were 0.06 and 128 μg/ml, respectively, suggesting that avilamycin displayed a potent antibacterial effect against anaerobic *C. perfringens*. The MIC distributions were brought into the ECOFFinder software, the cumulative distributions were calculated, and the cumulative distributions were fitted with nonlinear regression, the optimal fitting range was MIC ≤8 μg/ml. The calculated parameter values are shown in Table 2. The upper limit of the MIC distributions of *C. perfringens* in the 95% confidence interval was 0.25 μg/ml, defined as the CO_WT_.

**TABLE 2:** ECOFFinder analysis for avilamycin against *Clostridium perfringens*.

Parameters	Value
Selected Subset	≤8 μg/ml
Modal MIC	0.0625 μg/ml
Log_2_MIC Mode	−4
Max Log_2_MIC	8
Selected Log_2_ Mean	−4
Selected Log_2_ SD	1
95.0% Subset ECOFFs	0.25 μg/ml
97.5% Subset ECOFFs	0.25 μg/ml
99.0% Subset ECOFFs	0.5 μg/ml
99.5% Subset ECOFFs	0.5 μg/ml
99.9% Subset ECOFFs	1 μg/ml

MIC, minimum inhibitory concentration.

Note: Selected Subset was the optimal fitting range by nonlinear regression; Modal MIC was the most widely distributed MIC.

### 3.2 Pharmacokinetics–Pharmacodynamics Cutoff Value for Avilamycin Against *C. perfringens*

#### 3.2.1 Pharmacodynamics Study of *C. perfringens* HS42 *In Vitro* and *Ex Vivo*

**FIGURE 2:**
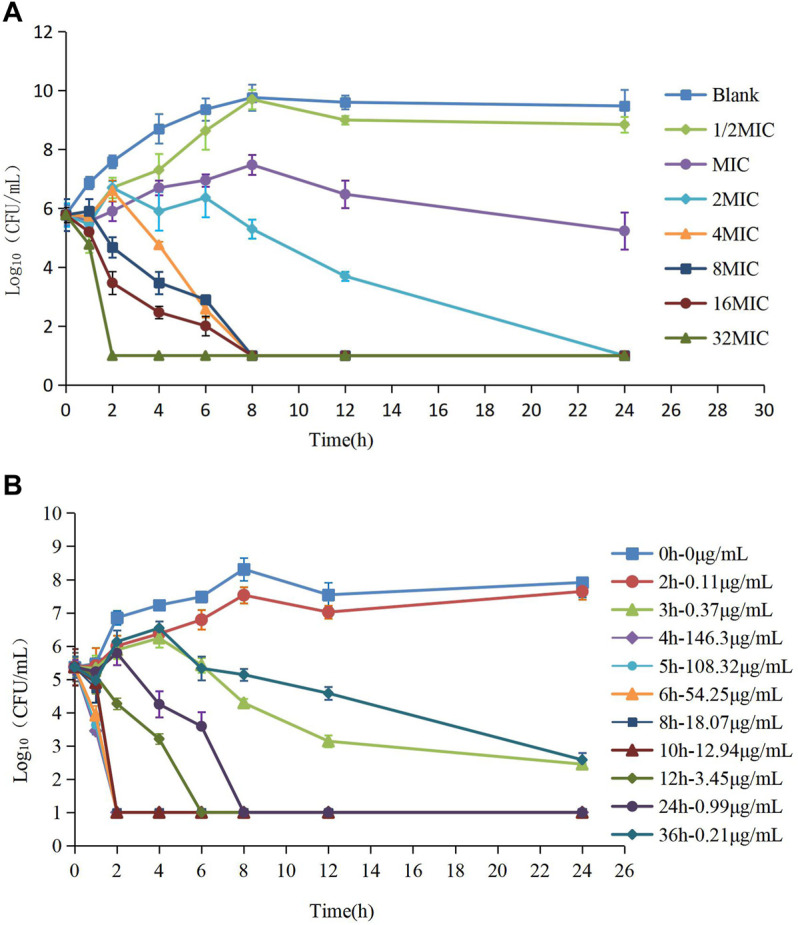
**(A,B)** The killing curves of avilamycin against *Clostridium perfringens in vitro* and *ex vivo*.

The MIC of HS42 was 0.25 μg/ml both in FT and ileal content, which indicated that the *ex vivo* antibacterial activity of avilamycin was the same as that *in vitro*. *In vitro* and *ex vivo* time-kill curves of the varying concentrations of avilamycin against HS42 are shown in Figure 2. For *in vitro* time-kill curves (Figure 2A), avilamycin can exert better bactericidal effect (≥2 MIC); all bacterium were eradicated without recovery growth and the bactericidal effect was enhanced with the increase of drug concentrations. According to *ex vivo* time-kill curves (Figure 2B), the ileal content concentrations from 4 to 24 h could eradicate bacterium completely. The curves were characteristically typical for concentration-dependent antibiotic activity both *in vitro* and *ex vivo*. The PK-PD index (AUC_0–24h_/MIC)_ex_ was selected to perform Monte Carlo simulation.

#### 3.2.2 Pharmacokinetics–Pharmacodynamics Integration Analysis

**FIGURE 3:**
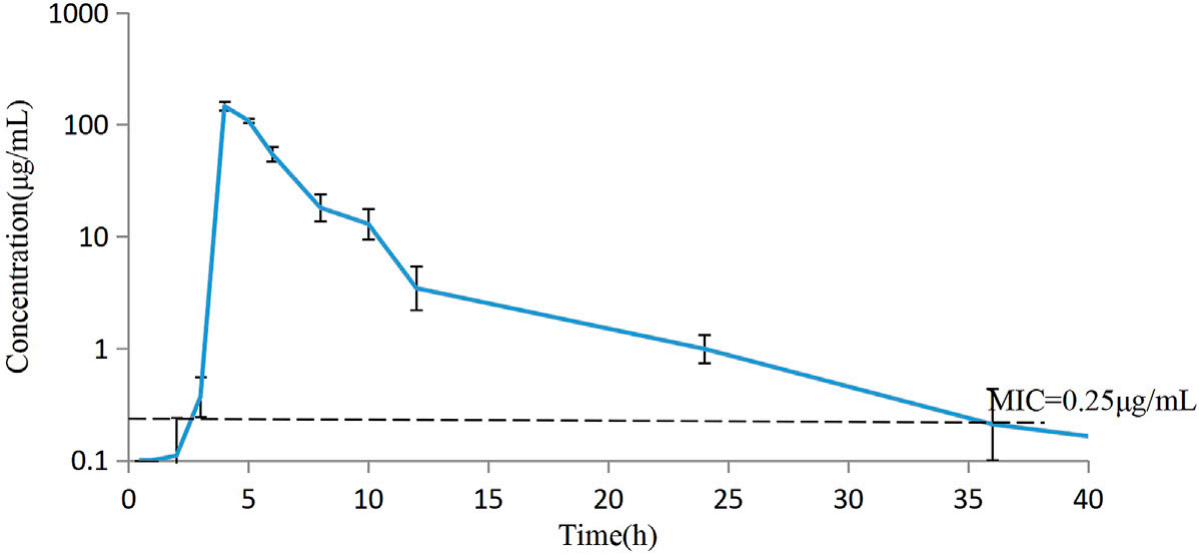
Mean concentration versus time curves for avilamycin in ileal content after oral administration at a dose of 4 mg/kg b.w. (*n* = 6).

The concentrations of avilamycin in the plasma and ileal content were greatly different. The drug concentrations in the plasma were below the LOD, indicating that the amount of avilamycin absorbed into the blood is very small, which is suitable for the treatment of gastrointestinal diseases. The drug concentrations in the ileal content are shown in Figure 3. The integrated PK parameters derived from the non-compartmental analysis of ileal content for avilamycin after oral administration at a dose of 4 mg/kg b.w. are shown in Table 3. The AUC_0–24h_ was 428.62 ± 14.23 h μg/mL, C_max_ was 146.30 ± 13.41 μg/ml, and T_max_ was 4 h. The PK study results showed that the concentration of avilamycin in the digestive tract after oral administration was higher than in the plasma, but it was released and eliminated faster in the ileal contents.

**TABLE 3:** Integrated PK parameters for avilamycin in ileal content after oral administration at a dose of 4 mg/kg b.w. (*n* = 6).

Parameters	Units	Values	SD
T_1/2λ_	h	3.27	1.08
C_max_	μg/mL	146.30	13.41
T_max_	h	4	—
AUC_0–24h_	H μg/mL	428.62	14.23
AUMC_0–24h_	h^2^ μg/mL	2596.87	151.83
MRT_0–24h_	H	5.25	0.38
CL/F	L/h/kg	0.01	0.0003

PK, pharmacokinetics; SD, standard deviation; C_max_, maximum concentration; T_max_, time of maximum concentration; T_1/2λz_, elimination half-life; AUC_0–24h_, area under the concentration curve; AUMC_0–24h_, first-order area under the concentration curve; MRT_0–24h_, mean residence time; CL/F, body clearance scaled by bioavailability.

The ileal content samples of 0, 2, 3, 4, 5, 6, 8, 10, 12, and 24 h after the oral administration were used to determine the *ex vivo* antibacterial activity of avilamycin against HS42. Table 4 shows the *ex vivo* concentrations and antibacterial effect obtained from the killing curves. The results showed that the PK-PD index (AUC_0–24h_/MIC)_ex_ reached higher ranges in maintaining antimicrobial efficacy. The relationship between the antimicrobial efficacy and *ex vivo* (AUC_0–24h_/MIC)_ex_ ratios was simulated by the inhibitory Sigmoid E_max_ equation, and the result is shown in Table 5. The values of (AUC_0–24h_/MIC)_ex_ at E = 0, −3, and −4 (bacteriostasis, bactericidal, and eradication) were 21.60, 36.15, and 53.24, respectively.

**TABLE 4:** *Ex vivo* concentration, (AUC_0–24h_/MIC)_ex_, and antibacterial effect obtained from the killing curves.

Time (h)	C_ex_ ^a^	(AUC0-24 h/MIC)ex^b^	Count 0 h (log10 CFU/mL)^c^	Count 24 h (log10 CFU/mL)^d^	E (log10 CFU/mL)^e^
0	0	0	5.37	7.91	2.54
2	0.11	10.56	5.37	7.64	2.27
3	0.37	35.52	5.37	2.45	−2.92
4	146.30	14,044.80	5.37	1	−4.37
5	108.32	10,398.72	5.37	1	−4.37
6	54.25	5208.00	5.37	1	−4.37
8	18.07	1734.72	5.37	1	−4.37
10	12.94	1242.24	5.37	1	−4.37
12	3.45	331.20	5.37	1	−4.37
24	0.99	95.04	5.37	1	−4.37
36	0.21	20.16	5.37	2.58	−2.79

a Drug concentrations at different times.

b *Ex vivo* PK–PD indexes at different times.

c Initial bacterial colonies incubated with different drug concentrations.

d Terminal bacterial colonies incubated with different drug concentrations after 24 h.

e Difference of antibacterial logarithm of ileal content samples incubated with the drug (d − c).

**TABLE 5:** *Ex vivo* pharmacodynamic parameters of avilamycin against *Clostridium perfringens*.

Parameters	Units	Values	SD
E_0_	Log_10_ CFU/mL	2.53	0.02
EC_50_	h	24.99	0.21
PD_max_	Log_10_ CFU/mL	6.91	0.01
Slope (N)	—	3.76	0.08
(AUC_0-24h_/MIC)_ex_ for bacteriostatic action (*E* =0)	h	21.60	—
(AUC_0-24h_/MIC)_ex_ for bactericidal action (*E* =−3)	h	36.15	—
(AUC_0-24h_/MIC)_ex_ for bacterial eradication action (*E* =−4)	h	53.24	—

#### 3.2.3 Monte Carlo Simulation and Pharmacokinetics–Pharmacodynamics Cutoff Value Determination

Based on AUC_0–24h_ data of 428.62 h g/mL and standard deviation of 14.23, Monte Carlo simulation was performed using Crystal Ball 7 software and AUC_0–24h_ of 10,000 pigs were generated by simulation. The PK-PD target (AUC_0–24h_/MIC)_ex_ for Monte Carlo simulation was 21.60, 36.15, and 53.24 h which attained inhibition, sterilization, and eradication effect.

**FIGURE 4:**
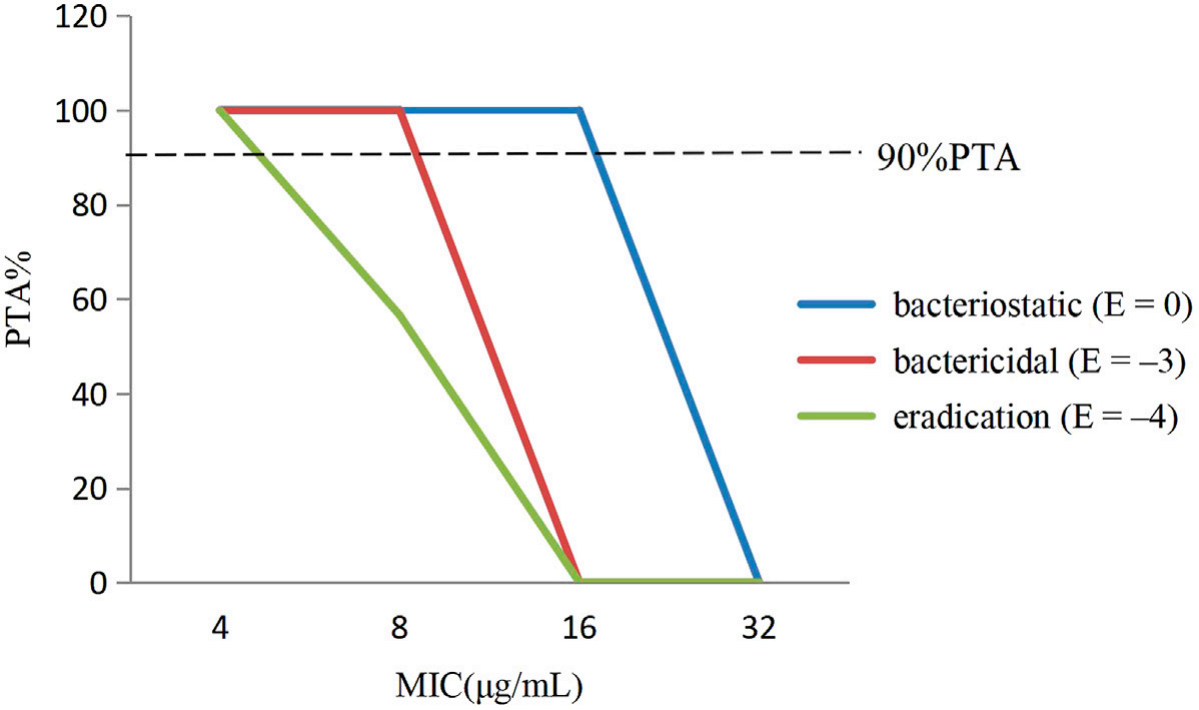
The probability of target attainment (%) of attaining [AUC_0–24h_/minimum inhibitory concentration (MIC)] ex ratio values by Monte Carlo simulation.

The PTA of avilamycin against *C. perfringens* under different antibacterial target was calculated based on the computation. For bacteriostatic action (E = 0), the PTA was 100% when the MIC was 8 μg/ml, while it was 0% when the MIC was 32 μg/ml. For bactericidal action (E = −3), the PTA ≥ 90% could be obtained for isolates with 8 ≤ MIC <16 μg/ml. For bacterial eradication action (E = −4), the PTA was calculated to be 56.59% at the MIC value of 8 μg/ml, but 100% at the MIC value of 4 μg/ml. The PTA was dependent on the MICs and PK-PD target. The larger the target values, the smaller the MIC required to achieve 90% compliance rate (Figure 4).

In this study, the PK-PD target of 36.15 h, which attained bactericidal activity when E = −3, was selected to determine the CO_PD_. Therefore, the CO_PD_ of avilamycin against swine *C. perfringens* was defined as MIC of 8 μg/ml, representing the bactericidal effects.

### 3.3 Dose Regimen

**FIGURE 5:**
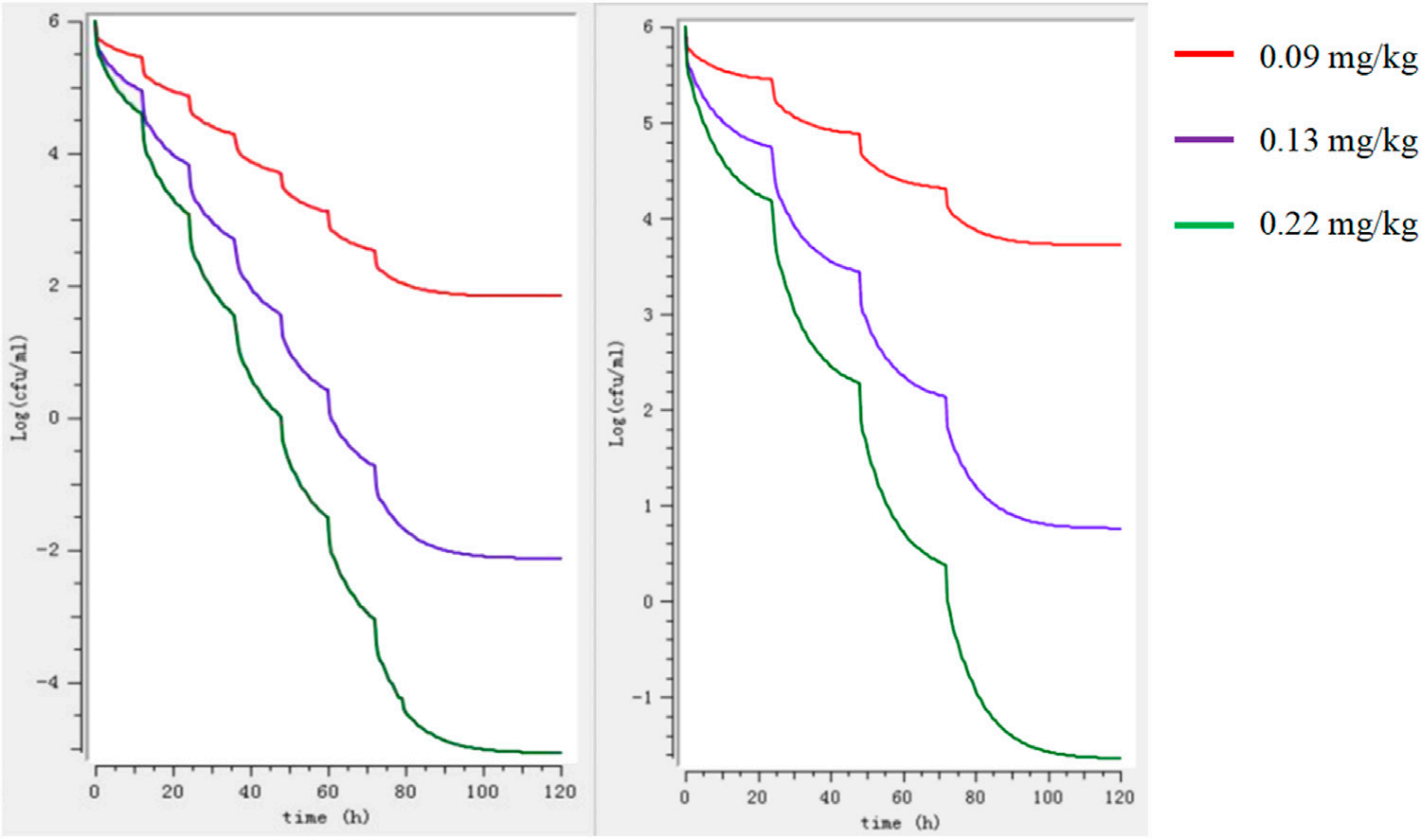
Model predictions of the growth of *Clostridium perfringens* at different doses regimens. Note: The left image shows the 12-h interval, and the right image shows the 24-h interval.

The binding rate of avilamycin in the ileal contents of pigs was 0.35, measured by the equilibrium dialysis method, and the free drug ratio (fu) of avilamycin in the ileal contents of pigs was 0.65. The doses required to achieve different antibacterial effects of avilamycin could be calculated by substituting the PK-PD index values into the dose equation. The preventive doses, therapeutic doses, and eradication doses of avilamycin mixed feeding were calculated to be 3.6, 5.2, and 8.8 mg/kg, respectively. The corresponding calculated preventive doses, therapeutic doses, and eradication doses were 0.09, 0.13, and 0.22 mg/kg, respectively. MlxPlore software was used to predict *C. perfringens* at different doses and dosing intervals (Figure 5). The results show that when daily doses are given at 12 h intervals, the expected effect can be achieved.

### 3.4 Clinical Cutoff Value for Avilamycin Against *C. perfringens*

The number of dead animals, recovered animals, and cured animals were counted during the treatment trial. Table 6 shows the data on the mortality rate, effective rate, and cure rate of each group. When the MIC was 0.06 μg/ml, the POC was 100%, and when the MIC was 0.25 μg/ml, the POC was 83.33%. Therefore, the range of CO_CL_ should be 0.06–0.25 μg/ml when the POC attained 90%.

**TABLE 6:** Probability of curve (POC) of avilamycin against *Clostridium perfringens* at different MICs.

Group	Strains (MIC)	Numbers	Number of dead animals	Dead rate	Effective rate	POC
Blank	—	6	0	0	—	—
Negative control	0.015 μg/ml	6	1	16.67%	—	—
	0.06 μg/ml	6	1	16.67%	—	—
	0.25 μg/ml	6	1	16.67%	—	—
	2 μg/ml	6	2	33.33%	—	—
	8 μg/ml	6	3	50%	—	—
Treatment group	0.015 μg/ml	6	0	0	100%	100%
	0.06 μg/ml	6	0	0	100%	100%
	0.25 μg/ml	6	1	16.67%	83.33%	83.33%
	2 μg/ml	6	1	16.67%	83.33%	83.33%
	8 μg/ml	6	2	33.33%	66.67%	50%

MIC, minimum inhibitory concentration.

Firstly, the CAR (AUC_succ_/AUC_total_) and MaxDiff calculation results are shown in Table 7. CAR could not be set at the lowest MIC or the highest MIC. If the CAR was increased with the MIC, If the CAR was increased with the MIC, then the second smallest CAR should be chosen as the final CAR. Based on this principle, the parameters MaxDiff and CAR were 0.22 and 0.85, respectively. The selection window of CO_CL_ was 0.06–2 μg/ml.

**TABLE 7:** Window of differing MaxDiff and CAR values.

MIC	Cure success	Numbers	%Success ≤ MIC	%Success > MIC	MaxDiff	AUC_Succ_	AUC_Total_	CAR
0.015	6	6	1	0.83	0.14	0.045	0.045	1
0.06	6	6	1	0.78	0.22	0.32	0.32	1
0.25	5	6	0.83	0.75	0.08	1.41	1.51	0.93
2	5	6	0.83	0.67	0.16	10.53	12.38	0.85
8	4	6	0.67	0.67	0	39.31	50.26	0.78

MIC, minimum inhibitory concentration.

Secondly, the NRA model with the highest coefficient (cubic) to simulate the corresponding model expression was *y* = 83.771–3.542x−4.24x^2^−0.054x^3^ (R = 0.94). According to the simulation expression, when POC was 90%, the Log_2_MIC was −2.17, and the equivalent MIC was 0.22 μg/ml. Thus, the recommended CO_CL_ was 0.06–0.22 μg/ml.

**FIGURE 6:**
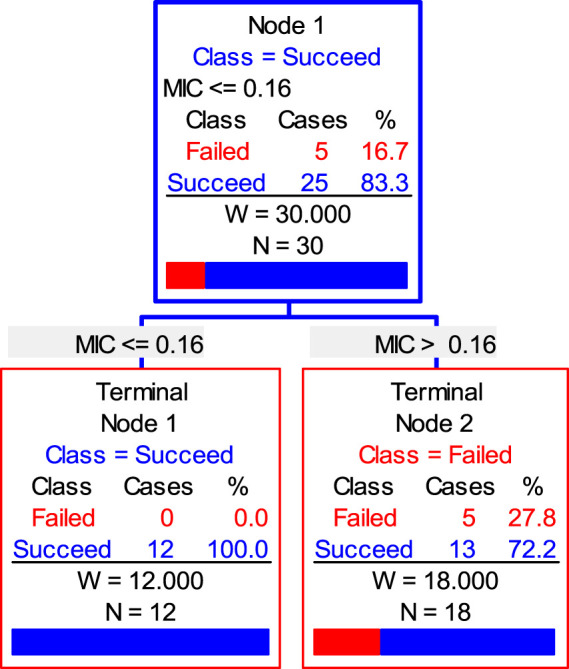
CART tree showing values of clinical outcome.

Finally, the data of the clinical trial (POC and MIC distributions) were brought into the Salford Predictive Modeler software for CART analysis. A regression tree was obtained as shown in Figure 6. From the regression tree, it could be seen that when MIC ≤0.16 μg/ml, the POC was 100%. When the MIC >0.16 μg/ml, the POC was 86.7%. When the POC equals to 90%, the MIC should be greater than 0.16 μg/ml but less than 0.25 μg/ml. Combined with the distributions, we chose the MIC nearing 0.22 and 0.16 μg/mL as the CO_CL_. Thus, the CO_CL_ for avilamycin against *C. perfringens* was 0.125 μg/ml.

### 3.5 Established Clinical Breakpoint

To sum up, the CO_WT_ was 0.25 μg/ml, the CO_PD_ was 8 μg/ml, and the CO_CL_ was 0.125 μg/ml. These three cutoffs were brought into the decision tree developed by the CLSI and were consistent with CO_PD_ > CO_WT_ > CO_CL_. Therefore, the CBP of avilamycin against *C. perfringens* was 0.25 μg/ml.

## 4 Discussion

*C. perfringens* is an environmental bacterium existing in the breeding industry and responsible for enteric disease with focal necrosis in the intestine. Also, it is a pathogen to public health through the food chain (Silva et al., 2009). Because of the antibacterial used for growth promotion or to prevent pathogenic effects of *C. perfringens*, extensive microorganism susceptibility researches have been conducted. Several studies showed that the *C. perfringens* antibiotic resistance was high to penicillin, bacitracin, tetracycline, and lincomycin (Akhi et al., 2015). But the oligosaccharide antimicrobial agent, avilamycin, has shown excellent activity against *C. perfringens*. The MICs of 30 *C. perfringens* isolates for avilamycin were tested in the local poultry industry, and the concentrations ranged from 4 to 512 μg/ml (Redondo et al., 2015). The MICs of avilamycin for 50 *C. perfringens* type A strains isolated from 1- to 7-day-old piglets, with or without diarrhea, ranged from 1 to 256 μg/ml, and the MIC with the highest number of strains was concentrated in 4 μg/ml (Salvarani et al., 2012).

In this study, the MIC distributions of avilamycin against the 120 clinical strains of *C. perfringens* ranged from 0.015 to 256 μg/ml, which had a wide scope than former research. Fitting the reported MIC data with our MIC distributions together, we got the same conclusion that the CO_WT_ was 0.25 μg/ml. The low MIC value (0.015 μg/ml) might be explained by regional disparities due to avilamycin not being popularly used in China. Meanwhile, the MIC distributions present a double peak, and there were a small number of resistant bacterium; the resistance mechanism of *C. perfringens* to avilamycin should be the focus and taken into account when establishing the CO_WT_ in future studies (Zheng et al., 2017). The mode of action of avilamycin is not well elaborated. It has been suggested that avilamycin acts by binding to the 30S part of the ribosome to inhibit bacterial protein synthesis (Wolf et al., 1973). However, a recent study indicated that resistance to avilamycin is associated with variations in the ribosomal protein L16 and thereby probably interacts with the peptidyl transferase activity. Some research showed that avilamycin binds to the 50S subunit, resulting in decreased susceptibility to avilamycin in bacteria (Mcnicholas et al., 2000).

We usually need to adopt certain statistical methods to define the CO_WT_ by the NRA (Hao et al., 2013; Smith et al., 2014). Conventionally, the MIC values were logarithmically transformed, and the cumulative distributions were subjected to regression simulation. The gradient was added to each fitting from the smallest distributed MIC to the most distributed MIC. The fit was kept between the optimal range between the actual values and estimated values, with a 95% confidence interval. Then the probability was predicted above the upper limit and below the lower limit, respectively, with the CO_WT_ as the upper limit of the wild-type distributions. In this experiment, the ECOFFinder program based on the NRA, was used to gradually replace and simplify the fitting process. We only needed to put the MIC distributions into the software to automatically fit and obtain CO_WT_.

PK-PD explored the relationship between the PK parameters and bacterial inhibition or killing and even extended it to the clinical outcome (Drusano, 2004). Over the last 30 years, a clear understanding of PK-PD on the many classes of antimicrobial agents had been researched, including the *β*-lactams, aminoglycosides, quinolones, macrolides, lincosamides, tetracyclines, and glycopeptides (Ambrose et al., 2007; Turnidge and Paterson 2007). In this study, we used healthy animals to establish the PK-PD model because the PK data were often generated in healthy representatives of the target species and the healthy animal model was more stable than the disease model, as well as showed better repeatability (CLSI 2007a). Furthermore, we suggest that the animal model can also be established in diseased animals at the same time and compared with healthy animal models if permitted.

The index T > MIC was used for time-dependent antibacterial agents, but the indexes AUC_0–24h_/MIC and C_max_/MIC were used for concentration-dependent antibacterial agents. The selected PK-PD index determined the PD parameter that best predicts efficacy in the animal model (Jr et al., 2007). The human medicine, evernimicin, which belonged to the same class as avilamycin, had been used to evaluate the bactericidal and sterilizing efficacies. Evernimicin exhibited a concentration-dependent killing kinetics, as the ratio AUC0–24h/MIC was the best index correlated with a reduction of bacterial count (Drusano et al., 2001). The bacteriostatic characteristic of avilamycin against *C. perfringens* indicated concentration dependence too. Hence, the PK-PD index (AUC_0–24h_/MIC) has been considered to calculate avilamycin *ex vivo* activity.

Most of the current studies on PK development have focused on the analysis of data in the plasma because these data are relatively stable and relatively easy to obtain. The concentrations of the drug at the target sites were generally estimated on the basis of the concentrations of the serum or plasma because the drug concentrations at the infection sites were largely correlated to the serum or plasma (Craig 1998; Ping et al., 2002). In our study, the drug concentrations in the blood might not accurately reflect the drug concentrations of the target tissue. For some digestive tract drugs, the T-shape ileal method had been used in swine for a more accurate PK study of the drugs in the digestive tract which could provide the target animals with maintaining a normal physiological state. Furthermore, researchers had shown that the measurement of free drug concentrations was more important than the total drug concentrations for evaluating the antimicrobial activity (Andes et al., 2002).

At present, the most common method for setting CO_CL_ was to collect a large number of clinical cases (Toutain et al., 2010; Lee et al., 2016), integrate the data, and finally use statistical analysis methods to obtain CO_CL_. Because of the limited clinical treatment of *C. perfringens* in pigs caused by avilamycin, the clinically challenged test had proceeded under laboratory conditions. The therapeutic dose adopted PK-PD recommended dosages which were linked to PK-PD with clinical treatment effect. The CO_CL_ formula POC = 1/[1 + e-a+bf (MIC)] proposed by EUCAST had not been applied in practice at present (Toutain et al., 2017). CO_CL_ reflected the upper limit of the MIC values associated with a high likelihood of clinical success (when POC equals to 90%). Based on this principle, five different MICs nearing MIC_50_, MIC_90_, CO_WT_, sensitive, and resistant strains were selected for conducting the clinical treatments. Three statistical analysis methods, including the “Window” approach, NRA, and CART analysis, were used to analyze the results of the clinical trials (Bhat et al., 2007; Cuesta et al., 2009; Cuesta et al., 2010; Turnidge and Martinez 2017), predicting the POC corresponding to the MIC distributions.

Comparing these three cutoff values, it was not difficult to find that the CO_PD_ was much higher than CO_WT_ and CO_CL_. Because the dose used in the PK-PD study was the recommended dose, which was a uniform dose for piglet diarrhea. *C. perfringens* was sensitive to avilamycin, and the drug was widely distributed in the digestive tract, leading to high AUC values and CO_PD_. Specially, when the CO_WT_ equaled CO_CL_, the CO_PD_ did not influence the final CBP. When the CO_WT_ did not equal CO_CL_, the CO_PD_ was used as a weighting factor, but the CBP would be established by the primary determinant CO_WT_ or CO_CL_ (CLSI 2007a). There was no clear methodology for establishing the CBP in veterinary medicine. In this study, we provided a train of thought and suggested that if there were obvious differences between CO_CL_, CO_WT_, and CO_PD_, the number of clinical samples should be expanded, and there is a need for expert subcommittee member deliberation on the relative weighting of these three cutoffs.

## 5 Conclusion

The rational use of antibiotics is becoming more and more important in veterinary clinics, and the abuse of antibiotics is the main reason for the development of bacterial resistance. After establishing three cutoff values in this study, the CBP of avilamycin against *C. perfringens* in swine was set to be 0.25 μg/ml, which can easily and clearly distinguish drug-resistant bacteria. At the same time, the findings of this study established that the optimal dosage of avilamycin for the treatment of pigs with *C. perfringens* infection is 5.2 mg/kg mixed feeding, and if the daily doses are given at 12 h intervals, the expected effect can be achieved.

## Data Availability

The original contributions presented in the study are included in the article/Supplementary Material, and further inquiries can be directed to the corresponding author.
